# Atrial Function Impairments after Pediatric Cardiac Surgery Evaluated by STE Analysis

**DOI:** 10.3390/jcm11092497

**Published:** 2022-04-29

**Authors:** Massimiliano Cantinotti, Pietro Marchese, Marco Scalese, Eliana Franchi, Nadia Assanta, Martin Koestenberger, Alessandra Pizzuto, Vitali Pak, Giuseppe Santoro, Vivek Jani, Shelby Kutty, Raffaele Giordano

**Affiliations:** 1 Fondazione G. Monasterio CNR-Regione Toscana, 54100 Massa, Italy; cantinotti@ftgm.it (M.C.); pitrino91@gmail.com (P.M.); efranchi@ftgm.it (E.F.); assanta@ftgm.it (N.A.); apizzuto@ftgm.it (A.P.); pakv2001@yahoo.com (V.P.); giuseppe.santoro@ftgm.it (G.S.); 2 Institute of Life Sciences, Scuola Superiore Sant’Anna, 56127 Pisa, Italy; 3 Institute of Clinical Physiology, 56124 Pisa, Italy; scalese@ifc.cnr.it; 4 Division of Pediatric Cardiology, Department of Pediatrics, Medical University Graz, 8036 Graz, Austria; martin.koestenberger@medunigraz.at; 5 Taussig Heart Center, Department of Pediatrics, Johns Hopkins Hospital, Baltimore, MD 21287, USA; vpjani@ucsd.edu (V.J.); shelby.kutty@gmail.com (S.K.); 6 Adult and Pediatric Cardiac Surgery, Department Advanced Biomedical Sciences, University of Naples “Federico II”, 80131 Naples, Italy

**Keywords:** congenital heart disease, STE echocardiography, atria, pediatric cardiac surgery

## Abstract

**Background:** Applications of atrial speckle tracking echocardiography (STE) strain (ε) analysis in pediatric cardiac surgery have been limited. This study aims to evaluate the feasibility of atrial STE ε analysis and the progression of atrial ε values as a function of post-operative time in children after pediatric cardiac surgery. **Methods:** 131 children (mean 1.69 ± 2.98; range 0.01–15.16 years) undergoing cardiac surgery were prospectively enrolled. Echocardiographic examinations were performed pre-operatively and at 3 different post-operative intervals: Time 1 (24–36 h), Time 2 (3–5 days), Time 3 (>5 days, before discharging). The right and left atrium longitudinal systolic contractile (Ct), Conduit (Cd), and Reservoir (R) ε were evaluated with a novel atrial specific software with both P- and R-Gating methods. One hundred and thirty-one age-matched normal subjects (mean 1.7 ± 3.2 years) were included as controls. **Results:** In all, 309 examinations were performed over the post-operative times. For each post-operative interval, all STE atrial ε parameters assessed were significantly lower compared to controls (all *p* < 0.0001). The lowest atrial ε values were found at Time 1, with only partial recovery thereafter (*p* from 0.02 to 0.04). All atrial ε values at discharge were decreased compared to the controls (all *p* < 0.0001). Significant correlations of the atrial ε values with cardio-pulmonary-bypass time, left and right ventricular ε values (*p* < 0.05), and ejection fraction (*p* < 0.05) were demonstrated. **Conclusions:** Atrial ε is highly reduced after surgery with only partial post-operative recovery in the near term. Our study additionally demonstrates that post-surgical atrial and ventricular ε responses correlated with each other.

## 1. Introduction

Speckle tracking echocardiography (STE)-derived myocardial strain (ε) analysis has demonstrated a significant prognostic value in pediatric cardiology [1,2,3,4]. Preliminary studies have reported both the feasibility and post-operative trends of left [4,5] and right [6] ventricular ε in children with congenital heart disease (CHD) after cardiac surgery. Atrial function has been shown to be an important predictor of cardiovascular outcomes in the adult population [1,2]. Atrial ε indices enable a better understanding of the overall function of the atrium, and there is increasing evidence to support the additional role of atrial ε as a sensitive parameter of ventricular diastolic dysfunction [1,2]. Investigation of STE-derived atrial ε [1,2] to evaluate post-surgical outcomes in pediatric cardiology has been limited. While some studies [7,8] evaluated differences in the atrial ε response a few months after percutaneous versus surgical closure of atrial septal defects on small cohorts (10–30 subjects), no large-scale analysis has been performed in the pediatric population [7,8].

The recent availability of pediatric nomograms for atrial [9,10,11] ε values, including those obtained with dedicated atrial strain software [12], may allow for the comparison of post-operative atrial ε values with normal values, consequently enabling further understanding of the degree of alteration in atrial mechanics. The primary aims of this investigation were to (1) assess the feasibility of atrial STE ε analysis in a large cohort of children after biventricular cardiac surgery, (2) evaluate the progression of STE derived atrial ε as a function of post-operative time, and (3) to compare these findings to atrial ε values in normal age-matched controls.

## 2. Methods

From May 2018 to May 2019, children undergoing biventricular cardiac surgery for CHD were prospectively enrolled at a Single Institution (Fondazione CNR-Regione Toscana, G. Monasterio, Massa, Italy). Demographic data are reported in Table 1. Echocardiographic examinations were performed at three different post-operative times according to institutional protocols: Time 1 (*n* = 131) 24–36 h, Time 2 (*n* = 108) 3–5 days, Time 3 (*n* = 70) 6–9 days, and at the immediate pre-operative time (*n* = 95). In complicated patients, echocardiograms were repeated whenever required for clinical management, and no examinations other than those necessary for treatment were performed. Only subjects in which atrial strain analysis was deemed feasible for at least 80% of the parameters evaluated were included in this study. A group of 131 age-matched normal subjects (mean 1.7 ± 3.2 years old) were used as controls from a pool of 721 healthy children collected in a previous study [9]. Echocardiograms were performed on Philips iE33 systems (Philips Medical Systems, Bothell, WA, USA) using 8 Mhz and 5 Mhz transducers [9,13]. All studies were performed with simultaneous electrocardiographic monitoring. Images were obtained in the apical four-chamber (4Ch), three-chamber (3Ch), and two-chamber (2Ch) views for the evaluation of left ventricular strain, and in apical four-chamber (4Ch) for the evaluation of right and left atrium speckle tracking analysis. The following parameters were calculated off-line: LA and RA longitudinal reservoir ε (SR), conduit ε (SCd.), and contractile ε (SCt) [9,13,14,15,16]. A dedicated atrial ε package was used for analysis on a computer workstation (QLAB 10; Philips Medical Systems, Andover, MA, USA) according to recent guidelines [9,10,11,12,13]: interatrial septum was included, and the atrial appendages were excluded. Feasibility is meant as the capacity of the software to recognize and define all the atrial wall segments. Atrial ε analysis was validated when at least ≥ 80% of the atrial segments were recognized correctly.

**Figure 1:**
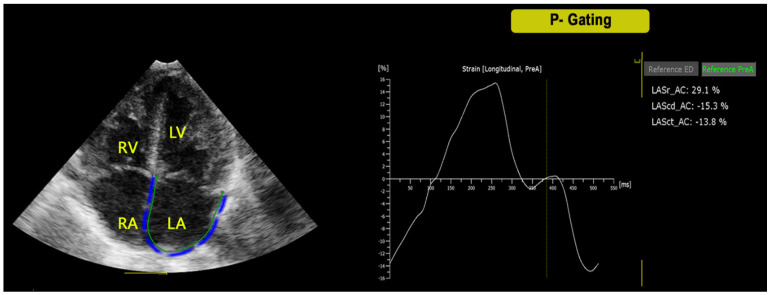
Left atrial ε analysis in the four-chamber view by using the atrial specific software for strain STE analysis with P-gating method. LA = Left atrium, LV = Left Ventricle, RA = Right Atrium, RV = Right Ventricle, Sr = strain reservoir, Sct = strain contractile, Scd = strain conduit.

**Figure 2:**
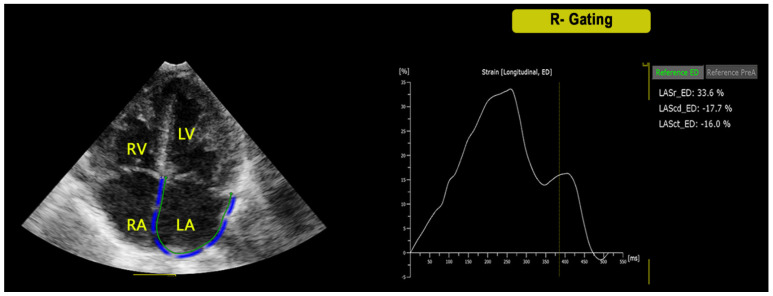
Left atrial ε analysis in the four-chamber view by using the atrial specific software for strain STE analysis with R-gating method. LA = Left atrium, LV = Left Ventricle, RA = Right Atrium, RV = Right Ventricle, Sr = strain reservoir, Sct = strain contractile, Scd = strain conduit.

After semi-automatic placement of basic markers (lateral and septal mitral/tricuspid annulus and septal roof) in end-diastole, the software automatically generated atrial contours and performed STE analysis in seven segments through the cardiac cycle [9]. Minimal manual adjustment of tracking was performed when required. For each parameter, the mean value of three consecutive measurements was obtained. The P waves (P-P gating) were used as the initiation of the ε calculation (Figure 1). The analysis was then repeated by using QRS complex (R-R gating) for the initiation of the ε calculation (Figure 2). End-diastole and onset of atrial contraction were checked and manually corrected according to mitral inflow profile. Two experienced pediatric cardiologists (M.C., E.F.) acquired the images and performed the measurements. Rates of intra-observer and inter-observer variability were calculated from 20 subjects, randomly selected.

The ejection fraction was calculated by the biplane Simpson method. Approval for this study was obtained from the Local Ethics Committee (Comitato Etico Meyer no. 62/2016). Parents or legal guardians of all the children were informed and provided written consent for participation in this study.

Images were acquired only in cooperative babies or in those who were sedated for clinical reason. No sedations just for image acquisition were performed. Only patients in sinus rhythm during the echocardiography were included in the present study.

All images and measurements were acquired by two independent and experienced pediatric cardiologists (M.C., E.F.). The low rate of intra- and inter-observer variability in atrial [9] ε measurements has been described in the previous reports [9].

### Statistical Analysis

All continuous variables and categorical variables were expressed as mean standard deviation (SD) and a number of cases and percentage, respectively. Comparison of continuous variables at different time points was performed using Wilcoxon test and nonparametric test for trends, as appropriate. Comparison of categorical variables at different time points was performed using a chi-square (Cochran–Armitage) test for trends in proportions. Comparison of age class was performed using a Mann–Whitney U test and a chi-square test as appropriate. Additionally, Pearson correlation coefficients (r) between strain values, operative data, and outcome parameters were determined. The control group of normal subjects was selected by 1:1 propensity score matching. Propensity score matching was calculated for each group with bivariate logistic regression analysis by age. All calculations were done by using SPSS v23 (SPSS Inc, Chicago, IL, USA) and STATA v13 software. A *p* < 0.05 was considered significant.

## 3. Results

### 3.1. Population

In all, 621 examinations were performed from May 2018 to May 2019 in 210 children (mean 2.25 ± 3.62 years; range 0.01–17.68 years). Seventy-nine patients were excluded for incomplete examinations (of these, 52 were not cooperative children and 27 had a poor acoustic window due to wounds and medications), leaving 309 examinations in 131 children (mean 1.69 ± 2.98 years; range 0.01–15.16 years) for final analysis. Among these children, 30 were neonates (0–31 days), 37 were infants (1–6 months), and 64 were >6 months. All demographic data are summarized in Table 1.

One hundred and thirty-one age-matched normal subjects (mean age 1.71 ± 2.94 years; range 0.03–14.12 years; mean BSA 0.43 ± 0.25) were included as controls. No differences in both age and BSA among healthy and CHD children were found (*p* = 0.94 and *p* = 0.657, respectively).

### 3.2. Feasibility

Feasibility, as assessed by the total number of studies from which relevant atrial ε parameters were acquired, ranged from 62 to 85% for all parameters. Feasibility was similar between all age groups, although at Time 1 and Time 2, it was marginally higher in neonates (≥80%) (*p* = 0.15 and *p* = 0.7). These results are summarized in Table 2.

### 3.3. Comparison vs. Normal Subjects

Pre-operative atrial ε values, obtained with either P-P or R-R gating methods, were significantly lower (*p* all < 0.0001), compared with normal subjects, with the only exception being the RA and LA contractile functions which were comparable with normal subjects.

### 3.4. The Post-Operative Trend for Atrial STE ε

**Figure 3:**
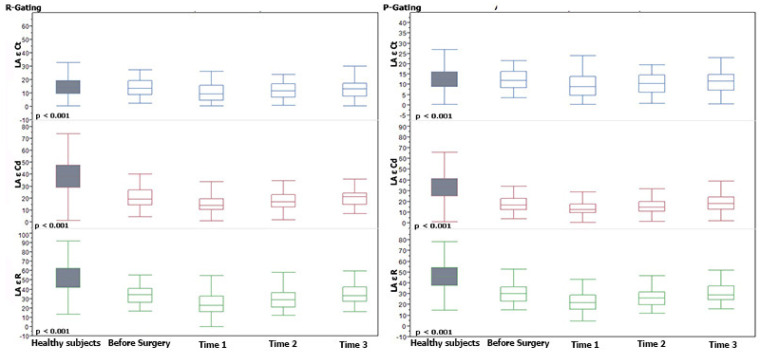
Left Atrial Strain trends at different pre-/post-operative times and in comparison to the control group. Median and interquartile range of ε values over time are shown. *p* values in the patient group were determined relative to strain from Time 1 to Time 3. Horizontal line = median; Box = interquartile range.

**Figure 4:**
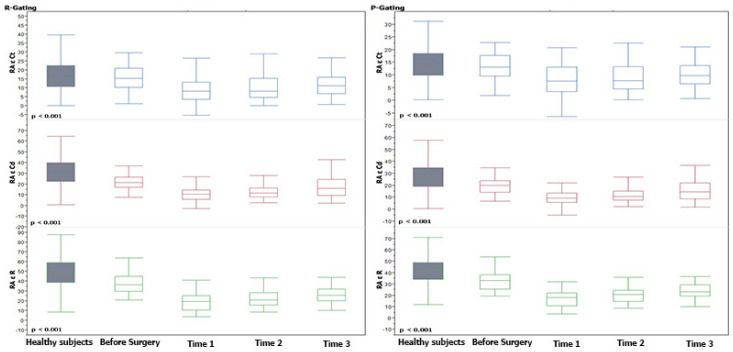
Right Atrial Strain trends at different pre-/post-operative times and in comparison to the control group. Median and interquartile range of ε values over time are shown. *p* values in the patient group were determined relative to strain from Time 1 to Time 3. Horizontal line = median; Box = interquartile range.

Post-operatively, all atrial ε values decreased with the lower values observed at Time 1 with a progressive recovery thereafter (*p* ranging from <0.0001 to 0.027). At discharge, however, all atrial ε parameters remained significantly lower compared to the control group (*p* ranging <0.0001 to 0.004), with the only exception being the LA Ct ε function, which was similar to healthy subjects’ values, as reported in Table 3. From Time 1 to Time 2, only the reservoir function, for both LA and RA, reported a significant increase (*p* from 0.007 to 0.022). From Time 2 to Time 3, the reservoir ε function, for the LA and RA, and the LA Cd ε function, reported a significant increase (*p* from 0.010 to 0.022). The remaining atrial functions showed a slower, however, significant, upward trend from Time 1 to Time 3 (*p* from 0.0009 to 0.002, *p* 0.0066 and 0.027, respectively). All these results are summarized in Table 3 and Supplementary Table S1, and Figure 3 and Figure 4.

### 3.5. Comparison of Post-Operative Trend for Atrial STE ε with Pre-Operative Values

Time 1 reported lower values in all LA and RA functions than the pre-operative data. Concerning Time 3, all LA ε values were totally recovered with values comparable with the pre-operative data (*p* > 0.05 each one), but, contrariwise, all RA ε values were still lower than the pre-operative data.

All these results are summarized in Table 3 and Supplementary Table S1.

### 3.6. Differences among P- and R-Gating Post-Operative ε Values

No significant differences in the post-operative time for the atrial **ε** trends were observed among values calculated with the P- and R-gating methods. Surgery atrial strain values calculated with P-gating, however, were constantly lower than those obtained with R-gating (*p* < 0.001).

### 3.7. Differences among Age Groups

Children younger than 6 months reported no significant differences from Time 1 to Time 2 in atrial STE ε with both gating methods. Children older than 6 months reported a significant increase from Time 1 to Time 2 only for the LA ε R function, through P-Gating (*p* 0.04), and for RA ε Cd through the two Gating methods, R and P (*p* 0.015 and *p* 0.017).

### 3.8. Correlation of Atrial Strain with Operative Data and Outcome Parameters

Strain parameters correlated with a CPB and cross-clamp time. At Time 1 (12–36 h post-surgery), CC inversely correlated with the RA conduit function in both the P- and R-gating methods (beta −0.04 *p* 0.009 and beta −0.004 *p* 0.045, respectively). Furthermore, a CPB is inversely related with the conduit function of both LA and RA (*p* all < 0.05), while it is inversely related with the reservoir function only for RA. Moreover, the LA reservoir function demonstrated an inverse correlation with the STAT-score (beta −3.78 *p* 0.008 for P-gating and beta −2.98 *p* 0.008 for R-gating).

No significant correlations were observed between the atrial STE ε values and the additional outcome parameters. Furthermore, no significant correlation was observed between the atrial STE values, body surface area (BSA), and age. These results are summarized in Supplementary Tables S2 and S3.

### 3.9. Correlation of Atrial Strain with Left and Right Ventricular STE

The atrial ε values correlated with the right/left ventricular ε and left ventricle ejection fraction (LVEF).

At Time 1 (12–36 h post-surgery), univariate regression analysis demonstrated that LV Longitudinal 4c, 2c, 3c, and global longitudinal (GL) ε values all positively correlated with all the LA longitudinal systolic ε with the R-gating method only (Supplementary Table S4). Right Ventricular ε, instead, did not correlate with LA and RA longitudinal systolic ε with both gating methods. Additionally, EF positively correlated only with LA ε R in both the R- and P-gating method (β = 0.28, *p* = 0.03 and β = 0.22, *p* = 0.03, respectively).

At Time 2, approximatively all atrial longitudinal systolic ε parameters positively related with all LV ε in the univariate regression analysis. Right Ventricular ε, instead, positively correlated only with RA ε R in both the R- and P-gating method (β = 0.44, *p* = 0.044 and β = 0.41, *p* = 0.023, respectively). Lastly, EF remained positively related with almost all atrial longitudinal systolic ε parameters. These results are summarized in Supplementary Table S4.

## 4. Discussion

In this study, we prospectively investigated atrial ε using STE and examined trends in atrial ε as a function of time after pediatric cardiac surgery. Defects assessed in this study included atrial septal defects, ventricular septal defects, atrioventricular septal defects, tetralogy of Fallot, transposition of the great arteries (TGA), and aortic stenosis. Our data demonstrate that STE-derived atrial ε analysis is feasible for various surgical indications of CHD across different ages.

By using a novel software specifically designed for atrial STE analysis [12], we report the feasibility of 62–85%, which is slightly lower than that presented in similar studies assessing ventricular ε analysis in a similar population (87–93% feasibility) [4,5]. Feasibility in this study was also lower than that observed for atrial ε analysis in a normal cohort (feasibility from 96.8% to 98.9%) [12].

Our study has demonstrated that all atrial ε parameters evaluated were significantly reduced compared to atrial ε in normal subjects [9]. As expected, the lowest atrial ε values were recorded at the first post-operative sample time (e.g., 12–36 h), with progressive recovery thereafter. However, at discharge, STE atrial ε values remained depressed compared to that observed in normal subjects (*p* < 0.0001). To our knowledge, this is the first study to assess the STE atrial ε response after pediatric cardiac surgery. Few studies have assessed atrial ε response in the medium-term for children undergoing percutaneous versus surgical closure of atrial septal defects [7,8]. Di Salvo and colleagues [7] evaluated color doppler LA and RA atrial ε in 15 subjects (mean age: 9 ± 3 years) 6 months after surgical ASD closure and compared these data with 15 age-matched patients who underwent percutaneous closure, along with 15 age-matched controls. In this particular study, patients who had undergone surgical AS closure had significantly lower LA and RA atrial ε compared to the percutaneous group and to normal subjects [7]. In another study, adults [8] (30 subjects; age 34.4 ± 8.3 years) > 1 year from surgical closure (range 1–5 years) were more likely to have an impaired RA strain and LA conduit strain compared to age-matched controls.

The trend of atrial ε response, we found, is similar to what has been described in the limited studies available for STE ventricular ε response after pediatric cardiac surgery [5,6,17]. In a study of over 117 children (mean age: 2.4 ± 3.9; range: 0–16 years), evaluated at different times pre-operatively and post-surgery, STE ε analysis revealed a significant LV systolic impairment after surgery with amelioration thereafter, but incomplete normalization at discharge [12]. In one retrospective study, De Boer et. al [17] showed in children (*n* = 204; median age: 3.7 ± 5.1 years) undergoing cardiac surgery for different CHD variants that LV and RV global longitudinal strain at the discharge echocardiography (median 7, range 6–14 days post-op) were significantly lower compared to age-matched controls (*n* = 78). Another study in 37 children (median age 19 months, interquartile range 5–63 months) demonstrated that RV peak systolic strain significantly decreased compared to the baseline (–10.5 ± 2.9% vs. –19.5 ± 4.8%; *p* < 0.0001). In this same study, RV peak systolic strain remained depressed compared with pre-operative values (–13.5 ± 4.0% vs. −9.5 ± 4.8%; *p* < 0.0001) [6]. The results from our study support these observations.

The degree of impairment in ε correlated with the duration of the cardio-pulmonary bypass and cross-clamp time. Our results support what has been published on ventricular ε response after pediatric cardiac surgery. In a study with over 33 children (4.2 ± 2.5 years) undergoing bypass cardiac surgery [5], LV longitudinal strain (ε) values were correlated to the aortic cross-clamp duration on post-operative day 0 (*r* = 0.47, *p* = 0.016) and post-operative day 1 (*r* = 0.53, *p* = 0.010) [5].

Of note, atrial ε values correlated with RV and LV ε, and LVEF. While similar correlations between atrial ε and LV strain [18,19] have been demonstrated in adult populations, data on children are lacking. In general, impaired atrial ε is more reflective of diastolic dysfunction [20,21]. The impaired atrial function observed in the present study may be indicative of abnormal ventricular diastolic properties. This is clinically relevant because traditional echocardiographic parameters for ventricular diastolic function assessment are poorly validated in children. Indeed, our data suggest that both the systolic and diastolic function are impaired after pediatric cardiac surgery, and that the dysfunction in the systole is correlated with dysfunction in the diastole.

We present data with a novel atrial specific software, calculated with both the P- and R-gating method. Although the R-wave method of gating is the one currently recommended, the P-gating method may be more appropriate for assessing the atrial function. Significant differences among the two methods have been demonstrated in healthy children where all atrial ε values were lower with P- than R-gating (*p* < 0.001) [9]. Similar differences were noted even in children with CHD after surgery, where atrial strain values calculated with P-gating were constantly lower than those obtained with R-gating. Feasibility and time trends of atrial strain values, however, did not change among the P- and R-gating methods. Thus, under a clinical point of view, both methods may be used; however, measurements obtained with the two different methods cannot be used interchangeably and, during the follow-up, values should be compared with those obtained with the same gating method (and with the same vendor) [13]. Atrial strain analysis with the novel semi-automatic software is very easy and fast, requiring just a few seconds for processing and automated reporting. Thus, considering data acquisition and data analysis, atrial strain analysis may be routinely introduced in the follow-up of children with CHD undergoing cardiac surgery, without big efforts or a loss of time.

### Limitations

We used STE software developed for the left atrium’s deformation only, which has been used for measuring right atrial ε. The use of vendor-specific software represents another limitation. Atrial ε was measured only in the four-chamber view, and not in the two- and three-chamber views [5,6]. Our study assessed longitudinal ε, including components of atrial contraction, reservoir, and conduit function, but did not include indices of atrial electromechanical coupling [1]. Examination times were slightly variable between patients depending on the institutional protocols for single CHD and the clinical indications. However, all 12–36 h examinations corresponded with the time of the first post-operative echocardiographic examination. Around half of the patients, including most older children assessed in our study, were extubated at the time of the examination, while most neonates and infants were intubated.

In addition, patients included in this investigation were heterogeneous and included a wide range of ages and cardiac defects. The relatively limited number of subjects enrolled did not allow for a sufficiently powerful sub-group analysis of patients (e.g., age, corrected vs. palliated, CHD groups), which may have constituted some bias in the final analysis. Nonetheless, despite the heterogeneity of the patient population assessed, significant trends in the ε response after cardiac surgery were observed, across different ages and different CHD variants. Age-related differences for LA and RA strain values were too limited and heterogenous to draw definitive conclusions. Parameter acquisition was incomplete at different sample times, reflecting an additional limitation. A comparison with pre-operative STE data is lacking; however, in uncorrected CHD, when significant shunts are present, the value of the ε data may be limited.

## 5. Conclusions

We report the progression of STE-derived atrial ε values after pediatric cardiac surgery as a function of the post-operative time. Our study observed that after pediatric cardiac surgery, all atrial ε parameters were significantly reduced compared to normal subjects. Atrial ε progressively recovered during the post-operative time; however, despite this improvement, atrial values remained significantly depressed compared with normal subjects upon discharge. The degree of atrial ε reduction seems to reflect the duration of the cardio-pulmonary bypass. Furthermore, the atrial and ventricular ε response appears to be correlated with each other. Additionally, the atrial ε response significantly correlated also with the left ventricular ejection fraction. Further studies in a larger cohort are required to validate and reinforce these observations.

## Figures and Tables

**Table 1:** Patients demographics.

	Older (nr 64)	Infant (nr 37)	Neonates (nr 30)	Total (nr 131)	
	Mean	Mean	Mean	Mean	*p*
Age (years)	3.23 ± 3.64	0.28 ± 0.13	0.03 ± 0.03	1.69 ± 2.98	<0.0001 *
BSA (m^2^)	0.57 ± 0.31	0.28 ± 0.06	0.21 ± 0.02	0.41 ± 0.28	<0.0001 *
CPB (min)	1.52 ± 0.75	1.6 ± 0.85	2.24 ± 1.16	1.69 ± 0.91	<0.0001 *
STAT-score	95.82 ± 46.38	93.97 ± 59.22	134.11 ± 99.79	103.96 ± 66.8	0.043 *
Extubation Time (days)	1.09 ± 1.22	2.74 ± 2.46	5.16 ± 5.77	2.48 ± 6.54	0.0389 *
ICU LOS (days)	10.19 ± 48.14	7.24 ± 7.15	10.67 ± 8.05	9.5 ± 34.75	0.023 *
Major complications	1 ^§^	2 °	3 *	6	
**CHD Numerosity**					
LVVO (nr)	18	17	2	37	
RVPO (nr)	20	9	1	30	
TGA (nr)	0	3	18	21	
LVPO (nr)	8	2	9	19	
RVVO (nr)	12	3	0	15	
AVSD (nr)	6	2	0	7	
Others (nr)	0	1	0	1	
Total	64	37	30	131	

AVSD = Atrioventricular Septal Defect; BSA = body surface area; CHD = congenital heart disease; CPB = cardio-pulmonary bypass; ICU = intensive care unit; LUS = lung ultrasound; LOS = length of stay; min = minutes; LVPO = Left Ventricle Pressure Overload; LVVO = Left Ventricle Volume Overload; RVPO = Right Ventricle Pressure Overload; RVVO = Right Ventricle Volume Overload; TGA = transposition of the great arteries; STAT-score = Society of Thoracic Surgeons/European Association of Cardio-Thoracic Surgery-STS/EACTS. ^§^ 1 tamponed, ° 1 tamponed, 1 Av block, * 1 AV block, 2 diaphragmatic paralyses.

**Table 2:** Feasibility at different post-operative times and in different age groups.

	Time 1			Time 2			Time 3		
%	Neonates	Infant	Older	Neonates	Infant	Older	Neonates	Infant	Older
LARε	80.0%	85.2%	72.1%	84.8%	80.0%	76.0%	70.4%	82.1%	61.9%
LACTε	80.0%	82.0%	72.1%	84.8%	80.0%	74.7%	70.4%	75.0%	61.9%
RARε	80.0%	78.7%	70.6%	75.8%	80.0%	77.3%	63.0%	75.0%	64.3%
RACTε	80.0%	75.4%	70.6%	75.8%	76.7%	74.7%	63.0%	67.9%	61.9%
Feasibility	80.0%	80.3%	71.3%	80.3%	79.2%	75.7%	66.7%	75.0%	62.5%

ε = strain, LAR = left atrial reservoir, LACT = left atrial contractile, RAR = right atrium reservoir, RACT = right atrium contractile.

**Table 3:** Mean and standard deviations of examinations at different pre-/post-operative times and in control group.

	Normal Subjects	Pre	Time 1	Time 2	Time 3
	Mean	Mean	Mean	Mean	Mean
(R-Gating) LA ε R	52.07 ± 15.87	35.21 ± 11.63	25.44 ± 12.17	29.94 ± 11.24	35.29 ± 13.57
(R-Gating) LA ε Cd	37.82 ± 13.8	20.85 ± 10.17	15.94 ± 8.48	18.21 ± 7.85	21.58 ± 9.23
(R-Gating) LA ε Ct	14.74 ± 7.27	13.95 ± 6.28	10.65 ± 7.46	12.31 ± 7.65	13.85 ± 8.79
(P-Gating) LA ε R	45.19 ± 13.03	30.72 ± 9.34	22.69 ± 9.62	26.46 ± 8.58	30.58 ± 10.21
(P-Gating) LA ε Cd	33.25 ± 12.57	18.37 ± 8.87	14.7 ± 7.92	16.41 ± 7.3	19.05 ± 7.82
(P-Gating) LA ε Ct	12.6 ± 5.41	12.36 ± 4.89	9.36 ± 5.89	10.61 ± 5.65	11.68 ± 6.45
(R-Gating) RA ε R	47.84 ± 16.6	38.49 ± 12.77	18.96 ± 9.49	22.4 ± 8.38	28.2 ± 14.71
(R-Gating) RA ε Cd	31.14 ± 13.66	22.72 ± 9.06	11.37 ± 6.72	13.49 ± 7.92	16.59 ± 10.27
(R-Gating) RA ε Ct	17.25 ± 9.09	15.84 ± 7.82	8.81 ± 6.64	10.19 ± 6.83	12.51 ± 7.86
(P-Gating) RA ε R	40.7 ± 13.15	33.22 ± 9.48	17,23 ± 7.92	20.26 ± 6.93	24.56 ± 11.13
(P-Gating) RA ε Cd	27.04 ± 12.4	19.9 ± 7.98	10.35 ± 6.55	12.64 ± 8.2	14.86 ± 9.22
(P-Gating) RA ε Ct	14.31 ± 6.52	13.38 ± 5.8	8.15 ± 5.72	8.98 ± 5.41	10.59 ± 5.8

ε = strain, R = reservoir, Ct = contractile, Cd = conduit.

## Data Availability

The data presented in this study are available on request from the corresponding author.

## Supplementary Materials

The following supporting information can be downloaded at: https://www.mdpi.com/article/10.3390/jcm11092497/s1, Table S1. Mean differences of examinations at different pre-/post-operative times and in control group. Table S2. Atrial STE *ε* mean values at different post-operative times for the entire cohort and in different age groups. Table S3. Correlations of Atrial STE ε with outcome predictors at Time 1. Table S4. Correlation between Atrial and ventricular STE ε at Time 1 and Time 2.
