# Supplementary material for: Atrial Function Impairments after Pediatric Cardiac Surgery Evaluated by STE Analysis
Source: J Clin Med. 2022 Apr 29;11(9):2497. doi: 10.3390/jcm11092497 (PMC9105784; doi:10.3390/jcm11092497)
Supplement: Supplementary file 1 [file jcm-11-02497-s001.zip › jcm-1661899-supplementary.pdf]

SUPPLEMENTAL TABLES

Supplementary Table S1. Mean differences of examinations at different pre-/post-operative times and in control group.

	Pre Vs T1	Pre Vs T2	Pre Vs T3	T1 Vs T2	T2 Vs T3	T1 Vs T3	Control Vs Pre	Control Vs T1	Control Vs T2	Control Vs T3
	p	p	p	p	p	p	p	p	p	p
**(R-Gating) LA ε R**	<0.0001*	0.0072*	0.97	0.0126*	0.0164*	<0.0001*	<0.0001*	<0.0001*	<0.0001*	<0.0001*
**(R-Gating) LA ε Cd**	0.0011*	0.08	0.69	0.07	0.028*	0.0003*	<0.0001*	<0.0001*	<0.0001*	<0.0001*
**(R-Gating) LA ε Ct**	0.0043*	0.17	0.94	0.15	0.29	0.0214*	0.4	<0.0001*	<0.0001*	0.4
**(P-Gating) LA ε R**	<0.0001*	0.0055*	0.94	0.007*	0.0146*	<0.0001*	<0.0001*	<0.0001*	<0.0001*	<0.0001*
**(P-Gating) LA ε Cd**	0.007*	0.15	0.67	0.144	0.022*	0.0017*	<0.0001*	<0.0001*	<0.0001*	<0.0001*
**(P-Gating) LA ε Ct**	0.001*	0.0183*	0.52	0.157	0.32	0.0292*	0.72	<0.0001*	<0.0001*	0.25
**(R-Gating) RA ε R**	<0.0001*	<0.0001*	0.0003*	0.022*	0.0103*	<0.0001*	<0.0001*	<0.0001*	<0.0001*	<0.0001*
**(R-Gating) RA ε Cd**	<0.0001*	<0.0001*	0.002*	0.08	0.08	0.0009*	<0.0001*	<0.0001*	<0.0001*	<0.0001*
**(R-Gating) RA ε Ct**	<0.0001*	<0.0001*	0.0379*	0.215	0.11	0.0066*	0.25	<0.0001*	<0.0001*	<0.0001*
**(P-Gating) RA ε R**	<0.0001*	<0.0001*	<0.0001*	0.0152*	0.015*	<0.0001*	<0.0001*	<0.0001*	<0.0001*	<0.0001*
**(P-Gating) RA ε Cd**	<0.0001*	<0.0001*	0.0042*	0.06	0.19	0.002*	<0.0001*	<0.0001*	<0.0001*	<0.0001*
**(P-Gating) RA ε Ct**	<0.0001*	<0.0001*	0.019*	0.37	0.15	0.027*	0.29	<0.0001*	<0.0001*	0.0004*

ε = strain, R= reservoir, Ct= contractile, Cd= conduit.

Supplementary Table S2. Atrial STE ε mean values at different post-operative times for the entire cohort and in different age groups.

	Pre		Time 1 (T1)		Time 2 (T2)		Time 3 (T3)		Pre Vs T1	Pre Vs T2	Pre Vs T3	T1 Vs T2	T1 Vs T3	T2 Vs T3
**Younger than 6 months**														
	**Mean**	**SD**	Mean	SD	Mean	SD	Mean	SD	p	p	p	p	p	p
**(R-Gating) LA ε R**	31.26	8.04	23.91	10.45	27.84	9.46	35.10	12.72	0.0019	0.12	0.18	0.08	0.0001	0.01
**(R-Gating) LA ε Cd**	16.95	7.55	15.73	7.72	16.75	7.18	19.83	8.57	0.5	0.91	0.19	0.5	0.04	0.1
**(R-Gating) LA ε Ct**	14.29	6.78	10.17	6.98	12.03	5.93	15.26	9.99	0.014	0.15	0.7	0.2	0.01	0.1
**(P-Gating) LA ε R**	27.34	6.62	21.72	8.51	24.85	7.34	30.03	9.61	0.003	0.16	0.2	0.08	0.0003	0.02
**(P-Gating) LA ε Cd**	15.08	7.13	14.83	7.83	15.25	7.10	17.42	7.63	0.9	0.92	0.24	0.8	0.2	0.24
**(P-Gating) LA ε Ct**	12.28	5.32	9.11	5.82	10.53	4.75	12.63	7.18	0.02	0.16	0.8	0.2	0.02	0.16
**(R-Gating) RA ε R**	34.35	9.67	18.19	10.28	22.25	9.14	26.32	15.42	<0.0001	<0.0001	0.04	0.09	0.02	0.25
**(R-Gating) RA ε Cd**	20.91	7.17	12.71	7.62	12.87	7.88	15.13	11.14	<0.0001	0.0002	0.04	0.9	0.32	0.4
**(R-Gating) RA ε Ct**	13.47	7.64	7.97	6.28	10.87	6.37	12.62	8.16	0.0023	0.17	0.7	0.06	0.02	0.4
**(P-Gating) RA ε R**	30.27	7.54	16.74	8.72	20.09	7.25	23.01	11.83	<0.0001	<0.0001	0.02	0.09	0.02	0.28
**(P-Gating) RA ε Cd**	18.68	6.81	11.44	7.48	12.03	8.39	13.80	10.33	0.0002	0.002	0.06	0.76	0.3	0.5
**(P-Gating) RA ε Ct**	11.61	5.96	7.68	5.69	9.62	4.93	10.55	6.09	0.01	0.2	0.5	0.14	0.08	0.6
**Older than 6 months**														
	Mean	SD	Mean	SD	Mean	SD	Mean	SD	p	p	p	p	p	p
**(R-Gating) LA ε R**	38.58	13.18	26.79	13.47	31.84	12.44	35.50	14.75	0.0001	0.03	0.4	0.7	0.013	0.4
**(R-Gating) LA ε Cd**	24.18	11.00	16.12	9.17	19.52	8.28	23.54	9.73	0.0004	0.04	0.8	0.07	0.002	0.9
**(R-Gating) LA ε Ct**	13.66	5.91	11.10	7.91	12.57	8.99	12.27	7.07	0.1	0.5	0.4	0.4	0.5	0.2
**(P-Gating) LA ε R**	33.59	10.40	23.54	10.51	27.91	9.41	31.20	11.03	<0.0001	0.015	0.4	0.04	0.005	0.4
**(P-Gating) LA ε Cd**	21.18	9.33	14.59	8.07	17.46	7.40	20.87	7.79	0.0008	0.06	0.9	0.08	0.002	0.8
**(P-Gating) LA ε Ct**	12.42	4.56	9.59	6.01	10.68	6.42	10.60	5.47	0.02	0.19	0.2	0.4	0.48	0.16
**(R-Gating) RA ε R**	41.76	14.06	19.64	8.80	22.53	7.83	29.91	14.17	<0.0001	<0.0001	0.004	0.13	0.0006	0.6
**(R-Gating) RA ε Cd**	24.15	10.19	10.18	5.64	14.01	8.03	17.93	9.48	<0.0001	<0.0001	0.03	0.02	<0.0001	0.5
**(R-Gating) RA ε Ct**	17.71	7.56	9.55	6.93	9.62	7.24	12.41	7.77	<0.0001	<0.0001	0.02	0.96	0.13	0.9
**(P-Gating) RA ε R**	35.54	10.30	17.66	7.22	20.41	6.75	25.97	10.52	<0.0001	<0.0001	0.002	0.08	0.0004	0.6
**(P-Gating) RA ε Cd**	20.86	8.78	9.39	5.52	13.14	8.12	15.83	8.21	<0.0001	0.0003	0.04	0.02	0.0003	0.5
**(P-Gating) RA ε Ct**	14.77	5.36	8.56	5.77	8.46	5.79	10.62	5.67	<0.0001	<0.0001	0.008	0.93	0.17	0.9

ε= strain, LAR=left atrial reservoir, LACT=left atrial contractile, RAR= right atrium reservoir, RACT=right atrium contractile.

Supplementary Table S3. Correlations of Atrial STE ε with outcome predictors at Time 1.

	CPB		CC		STAT		Aristotle		ETT	ICU LOS	Age	BSA
	**beta**	**p**	**beta**	p	beta	p	beta	p	p	p	p	p
**(R-Gating) LA ε R**		0.18		0.3	-3.78	0.008		0.06	0.8	0.8	0.97	0.4
**(R-Gating) LA ε Cd**	-0.03	0.02		0.07	-1.98	0.045	-1.1	0.039	0.7	0.9	0.8	0.6
**(R-Gating) LA ε Ct**		0.5		0.4		0.59		0.9	0.98	0.4	0.4	0.86
**(P-Gating) LA ε R**		0.09		0.2	-2.98	0.008	-1.2	0.04	0.7	0.7	0.9	0.38
**(P-Gating) LA ε Cd**	-0.02	0.03		0.1		0.14		0.06	0.7	0.9	0.9	0.72
**(P-Gating) LA ε Ct**		0.4		0.25		0.86		0.9	0.8	0.4	0.4	0.8
**(R-Gating) RA ε R**	-0.04	0.02		0.1		0.3		0.5	0.7	0.2	0.6	0.8
**(R-Gating) RA ε Cd**	-0.02	0.009	-0.04	0.045		0.31		0.67	0.1	0.8	0.5	0.5
**(R-Gating) RA ε Ct**		0.9		0.6		0.9		0.8	0.2	0.1	0.5	0.4
**(P-Gating) RA ε R**	-0.03	0.013		0.09		0.3		0.5	0.5	0.3	0.4	0.7
**(P-Gating) RA ε Cd**	-0.03	0.004	-0.04	0.009		0.08		0.8	0.4	0.8	0.4	0.6

**(P-Gating) RA ε Ct**	0.7	0.91	0.8	0.7	0.2	0.2	0.35	0.3

CPB=cardiopulmonary bypass, ICU LOS= intensive care long of stay, ETT=extubating time, ε= strain, LAR=left atrial reservoir, LACT=left atrial contractile, RAR= right atrium reservoir, RACT=right atrium contractile.

Supplementary Table S4. Correlation between Atrial and ventricular STE ε at Time 1 and Time 2.

	**LV 4 Ch ε**		**LV 2 Ch ε**		**LV 3 Ch ε**		**LVGL ε**		**RVGL ε**		**LV EF%**	
	**beta**	**p**	**beta**	**p**	**beta**	p	beta	p	beta	p	beta	p
						**At Time 1**						
**(R-Gating) LA ε R**	0.85	0.0007	0.99	0.0002	0.9	0.002	1.08	0.0002		0.6	0.28	0.03
**(R-Gating) LA ε Cd**	0.4	0.03	0.6	0.002	0.57	0.004	0.63	0.0016		0.7		0.13
**(R-Gating) LA ε Ct**		0.1		0.06		0.3		0.09		0.6		0.2
**(P-Gating) LA ε R**	0.66	0.0008	0.8	0.0002	0.72	0.0015	0.87	0.0001		0.7	0.22	0.03
**(P-Gating) LA ε Cd**		0.12	0.47	0.007	0.46	0.013	0.49	0.008		0.8		0.2
**(P-Gating) LA ε Ct**		0.2		0.1		0.45		0.17		0.54		0.16
**(R-Gating) RA ε R**		0.08		0.16		0.13		0.11		0.59		0.37
**(R-Gating) RA ε Cd**		0.99		0.57		0.44		0.51		0.17		0.57
**(R-Gating) RA ε Ct**		0.2		0.23		0.3		0.19		0.51		0.54
**(P-Gating) RA ε R**		0.14		0.27		0.18		0.19		0.47		0.52
**(P-Gating) RA ε Cd**		0.9		0.28		0.23		0.22		0.19		0.33
**(P-Gating) RA ε Ct**		0.25		0.32		0.45		0.26		0.61		0.73
						**At Time 2**						
**(R-Gating) LA ε R**	0.7	0.019	0.6	0.048		0.05	0.68	0.038		0.22	0.29	0.04
**(R-Gating) LA ε Cd**	0.46	0.02		0.16		0.28		0.13		0.8		0.23
**(R-Gating) LA ε Ct**		0.09		0.05	0.45	0.035	0.44	0.04		0.05	0.23	0.02
**(P-Gating) LA ε R**	0.56	0.01		0.05		0.07	0.52	0.037		0.3		0.05
**(P-Gating) LA ε Cd**	0.4	0.03		0.26		0.42		0.2		0.7		0.4
**(P-Gating) LA ε Ct**		0.06	0.31	0.03	0.35	0.02	0.36	0.03		0.05	0.18	0.0123
**(R-Gating) RA ε R**	0.66	0.004		0.07	0.72	0.004	0.66	0.011	0.44	0.044	0.3	0.018
**(R-Gating) RA ε Cd**	0.68	0.0017		0.2	0.72	0.002	0.64	0.0092		0.13		0.14
**(R-Gating) RA ε Ct**		0.21		0.06		0.15		0.12		0.08	0.2	0.047
**(P-Gating) RA ε R**	0.61	0.0014		0.06	0.65	0.0014	0.61	0.0048	0.41	0.023	0.25	0.0154
**(P-Gating) RA ε Cd**	0.64	0.0044		0.31	0.69	0.0046	0.6	0.0186		0.07		0.2
**(P-Gating) RA ε Ct**		0.19	0.31	0.045		0.15		0.11		0.07	0.16	0.049

ε= strain. LAR=left atrial reservoir, LACT=left atrial contractile, RAR= right atrium reservoir, RACT=right atrium contractile, LV= left ventricle, GL= global, RVGL= right ventricular global longitudinal strain, LV EF= left ventricle ejection fraction.
